# Preoperative factors improving the prediction of the postoperative sagittal orientation of the pelvis in standing position after total hip arthroplasty

**DOI:** 10.1038/s41598-020-72782-1

**Published:** 2020-09-29

**Authors:** Maximilian C. M. Fischer, Kunihiko Tokunaga, Masashi Okamoto, Juliana Habor, Klaus Radermacher

**Affiliations:** 1 Chair of Medical Engineering, Helmholtz-Institute for Biomedical Engineering, RWTH Aachen University, Aachen, Germany; 2 Niigata Hip Joint Center, Kameda Daiichi Hospital, Niigata City, Japan; 3 Department of Radiology, Kameda Daiichi Hospital, Niigata City, Japan

**Keywords:** Bone, Orthopaedics, Outcomes research, Musculoskeletal system

## Abstract

The aims of this study were to investigate if the sagittal orientation of the pelvis (SOP) in the standing position changes after total hip arthroplasty (THA) and evaluate what preoperative factors may improve the prediction of the postoperative standing SOP in the context of a patient-specific functional cup orientation. 196 primary THA patients from Japan were retrospectively selected for this study. Computed tomography imaging of the pelvis, EOS imaging of the lower body and lateral radiographs of the lumbar spine in the standing position were taken preoperatively. Common biometrics and preoperative Harris Hip Score were recorded. The EOS imaging in the standing position was repeated three months following THA. A 3D/2.5D registration process was used to determine the standing SOP. Thirty-three preoperative biometric, morphological and functional parameters were measured. Important preoperative parameters were identified that significantly improve the prediction of the postoperative standing SOP by using multiple linear LASSO regression. On average, the SOP changed significantly (*p* < 0.001) between the preoperative and postoperative standing position three months after THA by 3° ± 4° in the posterior direction. The age, standing lumbar lordosis angle (LLA) and preoperative supine and standing SOP significantly (*p* < 0.001) improve the prediction of the postoperative standing SOP. The linear regression model for the prediction of the postoperative standing SOP is significantly (*p* < 0.001) improved by adding the parameters preoperative standing SOP and LLA, in addition to the preoperative supine SOP, reducing the root mean square error derived from a leave-one-out cross-validation by more than 1°. The mean standing SOP in Japanese patients changes already three months after THA in comparison to the preoperative value. The preoperative factors age, LLA, supine and standing SOP can significantly improve the prediction of the postoperative standing SOP and should be considered within the preoperative planning process of a patient-specific functional cup orientation.

## Introduction

Functional component alignment in total hip arthroplasty (THA) is crucial to reduce the incidence of early revision surgery^1–5^. The functional cup orientation refers to the target alignment of the cup relative to the femoral component and the pelvic bone, reducing the risk of edge-loading, accelerated wear, impingement and dislocation for weight-bearing functional activities of daily living^6–10^. The functional cup orientation depends strongly on the sagittal orientation of the pelvis (SOP)^11^. The SOP is different among individuals and changes during activities of daily living^12–18^. The SOP in the standing position, at least, preferably also in the sitting position, should be considered during the planning of THA^19–22^. Multiple studies reported that the average preoperative change of the SOP from the supine to standing position ranges from 2° to 7° in the posterior direction^23^. However, the high inter-subject variability, that ranges from about 20° in the anterior or posterior direction, impedes the use of a unified value in the planning process^9,24,25^. Supine CT images can be used in navigated THA to reconstruct the osseous hip morphology for the preoperative planning process. Navigation systems or customized jigs enable an accurate intraoperative realization of the preoperative plan. The preoperative SOP in the standing position can be measured preoperatively^24,26–29^ and considered in the preoperative plan for the determination of the functional cup orientation. However, the measurement involves additional data acquisition in the planning process and might differ from the postoperative SOP in the standing position^25,30,31^. This leads to the following research question: Can preoperative biometric, morphological or functional parameters, in addition to the preoperative supine or standing SOP, improve the prediction of the postoperative standing SOP? This study investigates preoperative parameters that were reported to be possibly associated with or could, in the opinion of the authors, be related to the postoperative SOP in the standing position using an exploratory regression analysis. Thirty-three preoperative parameters were included as predictors in the analysis. They can be categorized into biometric, morphological and functional parameters. The biometric parameters include sex^25,32^, age^32^, height^33^, weight^33^ and body mass index (BMI)^33,34^. The morphological parameters are based on the sagittal geometry of the pelvis and are not affected by the pelvic orientation. They include angles, such as the pelvic incidence^35,36^ and sacral pubic angle^37^, and distances between osseous landmarks, such as the distance between the pubic symphysis or sacral center and the midpoint of the hip joint centers^36,38^. The functional parameters include the preoperative SOP in the standing and supine position, lumbar lordosis angle (LLA)^25^ and range of motion of the hip joint^39^.

## Patients and methods

### Patient data

All patients were informed about the use of their anonymized medical data, including medical images, for scientific research and written informed consent was obtained from all patients. The clinical protocol was approved by the Kameda Daiichi Hospital review board. The authors confirm that all methods were performed in accordance with the relevant guidelines and regulations.

Retrospectively, 201 consecutive patients were identified who had undergone unilateral primary THA between 2014 and 2017. Five patients with a fusion or ankylosis of more than two lumbar vertebrae were excluded from the study, resulting in a cohort of 196 patients (Table 1). Each patient received preoperatively supine CT imaging of the pelvis, standing orthogonal EOS imaging of the lower body and standing lateral radiographs of the lumbar spine. Additionally, the Harris Hip Score was collected. All patients underwent navigated THA using a Brainlab (Munich, Germany) or Stryker (Kalamazoo, MI, US) hip navigation system. All THAs were performed using an anterolateral modified Watson-Jones approach in the lateral position. Supine CT imaging was repeated six weeks and standing EOS imaging three months after surgery.

**Table 1:** Preoperative biometric parameters of all subjects and grouped into male and female.

	All (n = 196)	Male (n = 29)	Female (n = 168)	Two-sample t-test
Preoperative	Mean (SD, min. to max.)	Mean (SD, min. to max.)	Mean (SD, min. to max.)	*p* value
Age (years)	62.7 (10.7, 34.0 to 91.0)	63.5 (10.3, 38.0 to 83.0)	62.5 (10.8, 34.0 to 91.0)	0.651
Height (cm)	156.2 (7.8, 140.0 to 181.8)	166.2 (7.6, 151.1 to 181.8)	154.4 (6.4, 140.0 to 169.5)	< 0.001
Weight (kg)	57.1 (10.0, 35.9 to 103.8)	69.3 (12.0, 51.7 to 103.8)	55.0 (7.9, 35.9 to 82.5)	< 0.001
BMI (kg/m^2^)	23.3 (3.1, 16.6 to 34.5)	25.1 (3.7, 18.9 to 34.5)	23.1 (2.9, 16.6 to 31.2)	0.001

*p* value of the two-sample t-test between male and female (⍺ = 0.001).

### Pelvic parametrization

**Figure 1:**
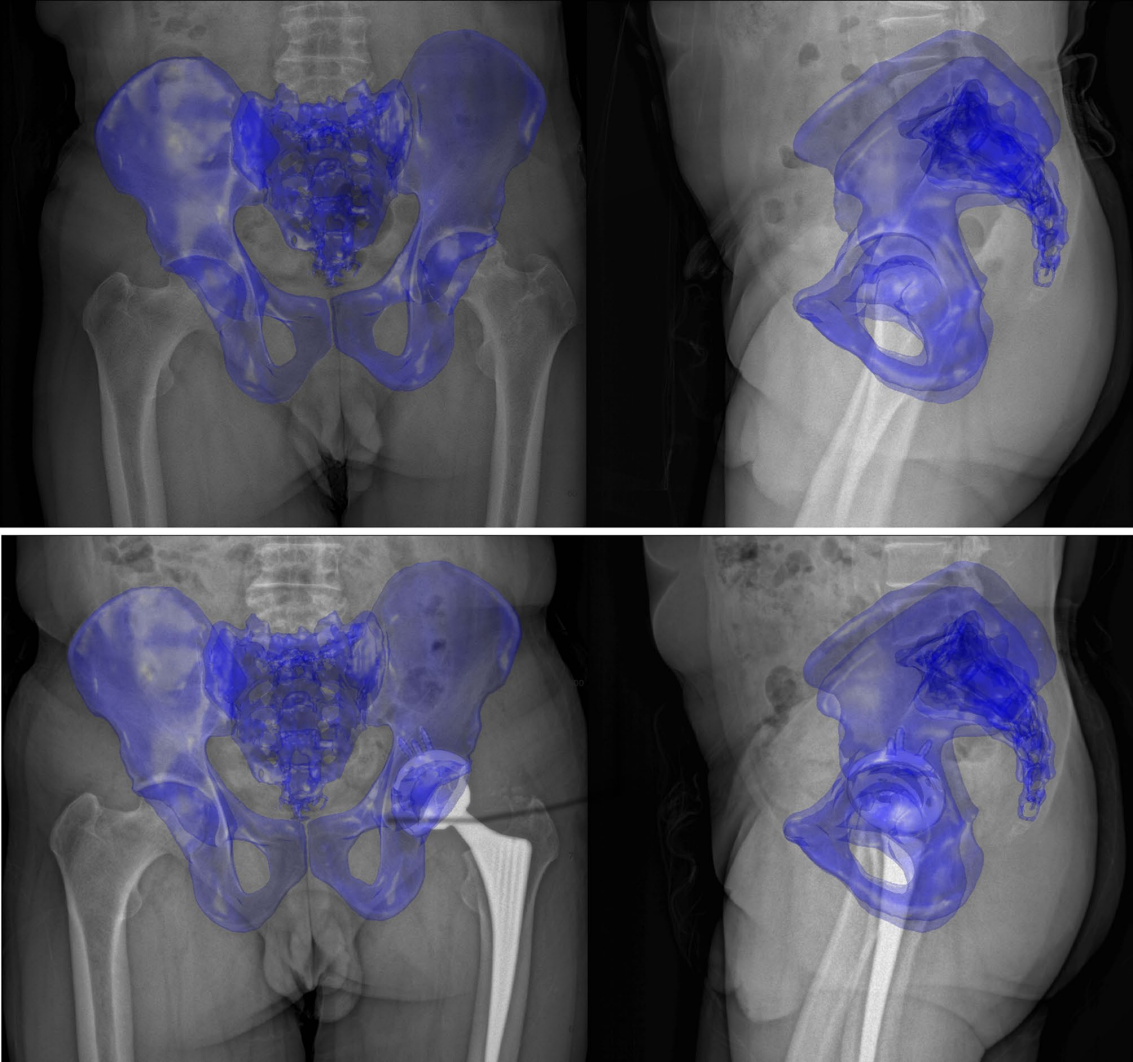
3D/2.5D registration of the surface model to the biplanar orthogonal EOS images using the CT2EOS method. The preoperative situation is depicted at the top. The postoperative situation, including the cup registered to the preoperative surface model, is depicted at the bottom.

The preoperative pelvic surface of each subject was reconstructed, as described in Fischer et al.^40^ The hip joint centers and osseous landmarks were identified using automatic methods after Cerveri et al.^41^ and Fischer et al.^40^ Each subject was reviewed for misdetections by one experienced examiner (M.C.M.F.). The landmarks were used to parametrize the pelvis and construct five common pelvic reference planes to measure the SOP. The definitions of the reference planes are described in the Supplementary Text S1. The postoperative hip bone was partially reconstructed, leaving out the region of the cup with metal artifacts. The partial reconstruction was registered to the preoperative hip bone using a rigid iterative closest point algorithm to determine the position of the reconstructed cup relative to the pelvis. The preoperative pelvic surface model including the registered cup was used for the 3D/2.5D registration to the postoperative EOS images (Fig. 1).

### Measurement of the SOP using the CT2EOS method

The hip joint centers, the pubic symphysis and the sacral promontory were manually selected on the EOS images in the standing position to pre-register the pelvic surface with the biplanar orthogonal EOS images, followed by a manual refinement of the registration by one experienced examiner (M.C.M.F.) (Fig. 1). Subsequently, the pre- and postoperative SOP were calculated in the supine and standing position based on the five different pelvic reference planes. The three-dimensional orientation of the planes in space was decomposed into the sagittal, frontal and transverse orientation of the pelvis: SOP, FOP and TOP.

#### Evaluation of the CT2EOS method

A trunk phantom (Kyoto Kagaku, Kyoto, Japan) was positioned in the EOS system using angle gauge blocks to control the ΔSOP and ΔFOP and a protractor disc to control the ΔTOP. Nineteen different orientations of the phantom were recorded. The SOP was measured using the CT2EOS and sterEOS (v1.6, EOS imaging, Paris, France) software. The measurement was repeated five times for each software by one experienced examiner (CT2EOS: M.C.M.F., sterEOS: M.O.). As physical measurements of the SOP would not be possible without damaging the phantom, the mean of the SOP measurements in the neutral position (ΔSOP = 0°, ΔFOP = 0°, ΔTOP = 0°) were used as reference value. The reference value and sagittal rotation added by the angle gauge blocks (ΔSOP) were subtracted from the measurements to compare both methods.

### Measurement of the LLA

The LLA is defined as the angle between the tangent lines along the vertebral body superior endplates of L1 and S1 and was measured on the preoperative lateral lumbar spine radiographs in the standing position. In the case of a L5-S1 fusion or ankylosis, the angle was measured between L1 and L5. Below, the term L5-S1 fusion also includes cases with L5-S1 ankylosis.

### Evaluation

The descriptive statistics are presented as mean (standard deviation (SD), minimum to maximum) or abbreviated as mean ± SD. All tests were two-sided with a significance level of 0.001. T-test or one-way analysis of variance (ANOVA) was used to identify significant differences between means.

The following thirty-three preoperative parameters were included as predictors in the regression analysis. The detailed definitions of all parameters are described in the Supplementary Text S1:

- Five biometric parameters: sex, age, height, weight, body mass index (BMI).
- Eighteen morphological parameters: Thirteen distances between different pelvic landmarks, five angles defined by pelvic landmarks. The morphological parameters are based purely on the shape of the pelvis and are independent of the position and orientation of the pelvis in space. All morphological parameters can be considered as sagittal pelvic parameters.
- Ten functional parameters: One of the five definitions of the preoperative SOP in the supine and standing position, the preoperative LLA in the standing position, a check for L5-S1 fusion, and six measures taken from the Harris Hip Score defining the range of motion.

The response variable was the postoperative SOP in the standing position three months after THA using the SOP definition corresponding to the predictors.

Two clinical scenarios were considered in the regression analysis regarding the preoperative image data available. Biometric parameters, morphological parameters, the preoperative supine SOP, the check for L5-S1 fusion and parameters from the Harris Hip Score were expected to be available in both scenarios.

(A) In this scenario, only CT images of the pelvis in the supine position are available. The scenario includes thirty-one preoperative parameters and excludes the preoperative standing SOP and the LLA in the standing position.
(B) In this scenario, CT images of the pelvis in the supine position and lateral imaging in the standing position are available. The scenario includes all thirty-three preoperative parameters.

Multiple linear LASSO regression^42^ with a leave-one-out cross-validation was performed to identify the optimal set of parameters for the prediction of the postoperative standing SOP. Parameters with a regression coefficient below 10^−3^ were excluded. Four models were compared:

1. The model with the maximum number of parameters (max model)
2. The model that corresponds to the minimum cross-validated mean squared error (MSE) (minMSE model)
3. The model that corresponds to one standard error (SE) of the minimum MSE (oneSE model)
4. The model that contains only the last nonzero coefficient (min model). This model contains only one predictor: The preoperative supine SOP for scenario A and the preoperative standing SOP for scenario B.

Likelihood ratio tests were used to evaluate whether the model including additional predictors performs significantly better than the model with fewer predictors. Additionally, the differences in the Akaike information criterion (ΔAIC)^43^ and the Bayesian information criterion (ΔBIC)^44^ are calculated, evaluating the level of support for the use of additional predictors. Moreover, the root mean square error (RMSE) in degrees calculated by a leave-one-out cross-validation and the adjusted coefficient of determination (R^2^_adj_) are reported for each model.

## Results

### Comparison of the CT2EOS and sterEOS software

Table 2 presents the results of the evaluation. The SOP was measured for three different reference planes: SSP, PTP and APP (see Supplementary Text S1). The CT2EOS method shows a superior accuracy over the sterEOS method of 0.1° ± 0.9°. Furthermore and in contrast to the sterEOS method, the accuracy of the CT2EOS method does not vary with the reference plane chosen. Both methods are affected by a rotation of the pelvis in the transverse plane (ΔTOP). However, this effect is considerably smaller for the CT2EOS method.

**Table 2:** Comparison of the CT2EOS and sterEOS software to measure the SOP.

(°)			CT2EOS			sterEOS		
			SOP_SSP_ − mean(SOP_SSP_(0,0,0)) − ΔSOP	SOP_PTP_ − mean(SOP_PTP_(0,0,0)) − ΔSOP	SOP_APP_ − mean(SOP_APP_(0,0,0)) − ΔSOP	SOP_SSP_ − mean(SOP_SSP_(0,0,0)) − ΔSOP	SOP_PTP_ − mean(SOP_PTP_(0,0,0)) − ΔSOP	SOP_APP_ − mean(SOP_APP_(0,0,0)) − ΔSOP
ΔSOP	ΔFOP	ΔTOP	Mean (SD, min. to max.)	Mean (SD, min. to max.)	Mean (SD, min. to max.)	Mean (SD, min. to max.)	Mean (SD, min. to max.)	Mean (SD, min. to max.)
0	0	− 30	0.3 (0.1, 0.1 to 0.5)	0.3 (0.1, 0.1 to 0.5)	0.3 (0.1, 0.1 to 0.5)	− 7.2 (0.6, − 7.7 to − 6.2)	− 1.6 (0.3, − 1.9 to − 1.2)	8.5 (1.3, 6.6 to 10.2)
0	0	− 15	0.2 (0.1, 0.1 to 0.3)	0.2 (0.1, 0.1 to 0.3)	0.2 (0.1, 0.1 to 0.3)	− 3.7 (0.7, − 4.9 to − 3.1)	− 0.9 (0.3, − 1.2 to − 0.5)	3.9 (0.6, 3.2 to 4.8)
0	0	0	0.0 (0.0, − 0.0 to 0.0)	− 0.0 (0.0, − 0.0 to 0.0)	− 0.0 (0.0, − 0.0 to 0.0)	− 0.0 (0.6, − 0.7 to 0.6)	0.0 (0.2, − 0.1 to 0.3)	− 0.0 (0.6, − 0.9 to 0.5)
0	0	15	0.5 (0.0, 0.5 to 0.5)	0.5 (0.0, 0.5 to 0.5)	0.5 (0.0, 0.5 to 0.5)	− 4.4 (0.3, − 4.8 to − 4.1)	− 1.3 (0.4, − 1.6 to − 0.8)	0.9 (0.3, 0.3 to 1.1)
0	0	30	0.6 (0.0, 0.6 to 0.6)	0.6 (0.0, 0.6 to 0.6)	0.6 (0.0, 0.6 to 0.6)	− 7.1 (0.6, − 7.9 to − 6.4)	− 2.6 (0.3, − 2.9 to − 2.2)	9.2 (3.1, 4.3 to 11.9)
15	0	− 30	− 0.9 (0.6, − 1.8 to − 0.5)	− 0.9 (0.6, − 1.9 to − 0.5)	− 0.9 (0.6, − 1.9 to − 0.5)	− 7.2 (1.0, − 8.8 to − 6.0)	− 2.6 (0.4, − 3.2 to − 2.2)	6.4 (2.9, 3.7 to 10.1)
15	0	− 15	0.6 (0.5, 0.0 to 1.0)	0.6 (0.5, 0.0 to 1.0)	0.6 (0.5, 0.0 to 1.0)	− 3.7 (0.4, − 4.0 to − 3.1)	− 0.3 (0.4, − 0.9 to 0.1)	2.9 (0.4, 2.3 to 3.3)
15	0	0	− 0.1 (0.4, − 0.3 to 0.7)	− 0.1 (0.4, − 0.3 to 0.7)	− 0.1 (0.4, − 0.3 to 0.7)	− 2.7 (0.4, − 3.2 to − 2.2)	− 0.5 (0.4, − 0.8 to 0.0)	− 1.1 (0.3, − 1.4 to − 0.8)
15	0	15	− 1.1 (0.4, − 1.3 to − 0.4)	− 1.1 (0.4, − 1.3 to − 0.3)	− 1.1 (0.4, − 1.3 to − 0.3)	− 5.5 (0.7, − 6.4 to − 4.6)	− 2.5 (0.2, − 2.7 to − 2.3)	0.3 (0.4, − 0.1 to 0.9)
15	0	30	− 2.1 (0.2, − 2.5 to − 2.0)	− 2.1 (0.2, − 2.5 to − 2.0)	− 2.1 (0.2, − 2.4 to − 2.0)	− 9.2 (1.4, − 11.2 to − 7.4)	− 5.7 (0.7, − 6.5 to − 4.8)	1.7 (1.4, 0.1 to 3.8)
− 15	0	− 30	1.6 (0.9, 0.7 to 2.5)	1.6 (0.9, 0.7 to 2.5)	1.6 (0.9, 0.7 to 2.5)	− 6.8 (1.5, − 8.2 to − 4.5)	0.5 (0.3, 0.2 to 0.9)	8.9 (0.8, 7.6 to 9.4)
− 15	0	− 15	0.2 (0.0, 0.2 to 0.3)	0.3 (0.0, 0.2 to 0.3)	0.2 (0.0, 0.2 to 0.3)	− 3.5 (0.6, − 4.1 to − 2.9)	− 1.3 (0.3, − 1.7 to − 1.0)	13.1 (0.9, 12.0 to 14.2)
− 15	0	0	− 0.8 (0.5, − 1.2 to − 0.2)	− 0.8 (0.5, − 1.2 to − 0.2)	− 0.8 (0.5, − 1.2 to − 0.2)	− 2.3 (0.4, − 2.8 to − 1.7)	− 1.3 (0.1, − 1.5 to − 1.2)	− 2.0 (0.5, − 2.7 to − 1.3)
− 15	0	15	0.4 (0.4, 0.1 to 1.1)	0.4 (0.4, 0.1 to 1.1)	0.3 (0.4, 0.1 to 1.1)	− 4.6 (0.9, − 5.4 to − 3.3)	− 1.5 (0.4, − 1.9 to − 0.8)	0.6 (0.2, 0.4 to 0.9)
− 15	0	30	1.5 (0.0, 1.4 to 1.5)	1.4 (0.0, 1.4 to 1.5)	1.4 (0.0, 1.4 to 1.5)	− 7.1 (0.5, − 8.0 to − 6.6)	− 1.7 (0.3, − 2.1 to − 1.4)	11.5 (1.1, 10.3 to 13.2)
15	15	0	0.2 (0.9, − 0.4 to 1.6)	0.2 (0.9, − 0.4 to 1.7)	0.2 (0.9, − 0.4 to 1.7)	− 2.3 (0.8, − 3.4 to − 1.6)	− 0.7 (0.2, − 0.9 to − 0.5)	− 2.0 (0.6, − 2.9 to − 1.4)
15	15	0	0.8 (0.5, 0.6 to 1.6)	0.8 (0.5, 0.6 to 1.6)	0.8 (0.5, 0.5 to 1.6)	− 3.5 (1.1, − 4.7 to − 1.8)	− 2.2 (0.4, − 2.8 to − 1.7)	− 0.5 (0.4, − 1.0 to 0.0)
30	0	0	0.8 (0.0, 0.8 to 0.8)	0.8 (0.0, 0.8 to 0.8)	0.8 (0.0, 0.8 to 0.8)	− 1.4 (0.6, − 2.1 to − 0.5)	− 0.1 (0.1, − 0.3 to 0.1)	− 0.9 (0.3, − 1.3 to − 0.5)
− 30	0	0	0.1 (0.4, − 0.1 to 0.9)	0.1 (0.4, − 0.1 to 0.9)	0.1 (0.4, − 0.1 to 0.9)	− 2.3 (0.6, − 3.0 to − 1.3)	− 0.9 (0.6, − 1.9 to − 0.4)	− 2.5 (1.6, − 4.4 to − 0.6)
All values			0.1 (0.9, − 2.5 to 2.5)	0.1 (0.9, − 2.5 to 2.5)	0.1 (0.9, − 2.4 to 2.5)	− 4.4 (2.5, − 11.2 to 0.6)	− 1.4 (1.4, − 6.5 to 0.9)	3.1 (5.0, − 4.4 to 14.2)
All absolute values			0.7 (0.6, 0.0 to 2.5)	0.7 (0.6, 0.0 to 2.5)	0.7 (0.6, 0.0 to 2.5)	4.5 (2.4, 0.2 to 11.2)	1.5 (1.3, 0.0 to 6.5)	4.1 (4.2, 0.0 to 14.2)

The measurement was repeated five times for each software by one examiner. The mean of the measurements of the phantom in the neutral position (ΔSOP = 0°, ΔFOP = 0°, ΔTOP = 0°) and ΔSOP were subtracted from each measurement for the comparison.

### Prediction of the postoperative standing SOP

There were no significant differences in the change of the SOP among the five different definitions of the SOP either from the preoperative supine position (*p* = 0.96, ANOVA) or from the preoperative standing position (*p* = 0.96, ANOVA) to the postoperative standing position. Consequently, the sacral slope (SOP_SSP_) was selected from the five definitions to represent the SOP exemplarily hereafter. The results of the other four definitions and descriptive statistics of each parameter are presented in the Supplementary Tables S2. Table 3 shows the significant change of the SOP_SSP_ from the preoperative supine and standing position to the postoperative standing position three months after THA.

**Table 3:** Changes of SOP_SSP_.

	Mean (SD, min. to max.)	Mean (SD, min. to max.)	Mean (SD, min. to max.)	*p* value
	Preoperative supine	Postoperative standing	Change	
SOP_SSP_ (°)	38.5 (8.3, 15.0 to 57.3)	34.7 (10.2, 1.0 to 58.5)	− 3.8 (5.2, − 23.9 to 12.4)	< 0.001
	Preoperative standing	Postoperative standing	Change	
SOP_SSP_ (°)	37.7 (9.8, 8.4 to 63.3)	34.7 (10.2, 1.0 to 58.5)	− 3.0 (4.1, − 15.7 to 7.1)	< 0.001

*p* value of the paired-sample t-test between the preoperative and postoperative SOP_SSP_.

Regarding both scenarios of the postoperative standing SOP_SSP_ prediction, the max model does not significantly improve the minMSE model, whereas the minMSE model significantly improves the oneSE model and the oneSE model performs significantly better than the min model (*p* < 0.001, likelihood ratio test). The ΔAIC shows moderate support for the minMSE model (ΔAIC > 4.6) and very strong support for the oneSE model (ΔAIC > 13.8). Moreover, the positive ΔBIC indicates the use of the oneSE model (Table 4).

**Table 4:** Comparison among the four models of both scenarios for the prediction of the postoperative standing SOP_SSP_ including the number of predictors (NoP) and the *p* value of the likelihood ratio test.

Scenario A							Scenario B						
Model	NoP	RMSE (°)	R^2^_adj_	ΔAIC	ΔBIC	*p* value	Model	NoP	RMSE (°)	R^2^_adj_	ΔAIC	ΔBIC	*p* value
max	28	4.97	0.802	− 10.9	− 30.5	0.98	max	30	3.72	0.887	− 11	− 34	0.887
minMSE	22	4.81	0.808	6.4	− 59.2	< 0.001	minMSE	23	3.61	0.89	6	− 59.6	< 0.001
oneSE	2	4.82	0.782	29.6	26.4	< 0.001	oneSE	3	3.65	0.875	45	38.5	< 0.001
min	1	5.2	0.745				min	1	4.08	0.841			

The models of scenario B including the preoperative standing parameters perform significantly better than the models of scenario A without the preoperative standing parameters (*p* < 0.001, likelihood ratio test).

Table 5 shows the detailed oneSE models of both scenarios. For scenario A, the oneSE model contains the biometric parameter age in addition to the preoperative supine SOP_SSP_ for the prediction of the postoperative standing SOP_SSP_. An increase of age of one year would reduce the postoperative standing SOP_SSP_ by 0.19°. An increase of the preoperative supine SOP_SSP_ of 1° would increase the postoperative standing SOP_SSP_ by 1.01°. For scenario B, the oneSE model contains the functional parameters LLA and preoperative supine SOP_SSP_ in addition to the preoperative standing SOP_SSP_ for the prediction of the postoperative standing SOP_SSP_. An increase of the LLA, preoperative supine SOP_SSP_ or preoperative standing SOP_SSP_ of 1° would increase the postoperative standing SOP_SSP_ by 0.09°, 0.31° or 0.61°, respectively.

**Table 5:** Parameters of the oneSE model of both scenarios for the prediction of the postoperative standing SOP_SSP_.

Scenario A			Scenario B		
Parameter	Estimate (SE)	*p* value	Parameter	Estimate (SE)	*p* value
(Intercept)	7.53 (2.85)	0.009	(Intercept)	− 4.59 (1.25)	< 0.001
Age	− 0.19 (0.03)	< 0.001	LLA	0.09 (0.02)	< 0.001
Preoperative supine SOP_SSP_	1.01 (0.04)	< 0.001	Preoperative supine SOP_SSP_	0.31 (0.06)	< 0.001
			Preoperative standing SOP_SSP_	0.61 (0.05)	< 0.001

The results of the other four definitions of the SOP are very similar and are presented in the Supplementary Tables S2.

## Discussion

**Figure 2:**
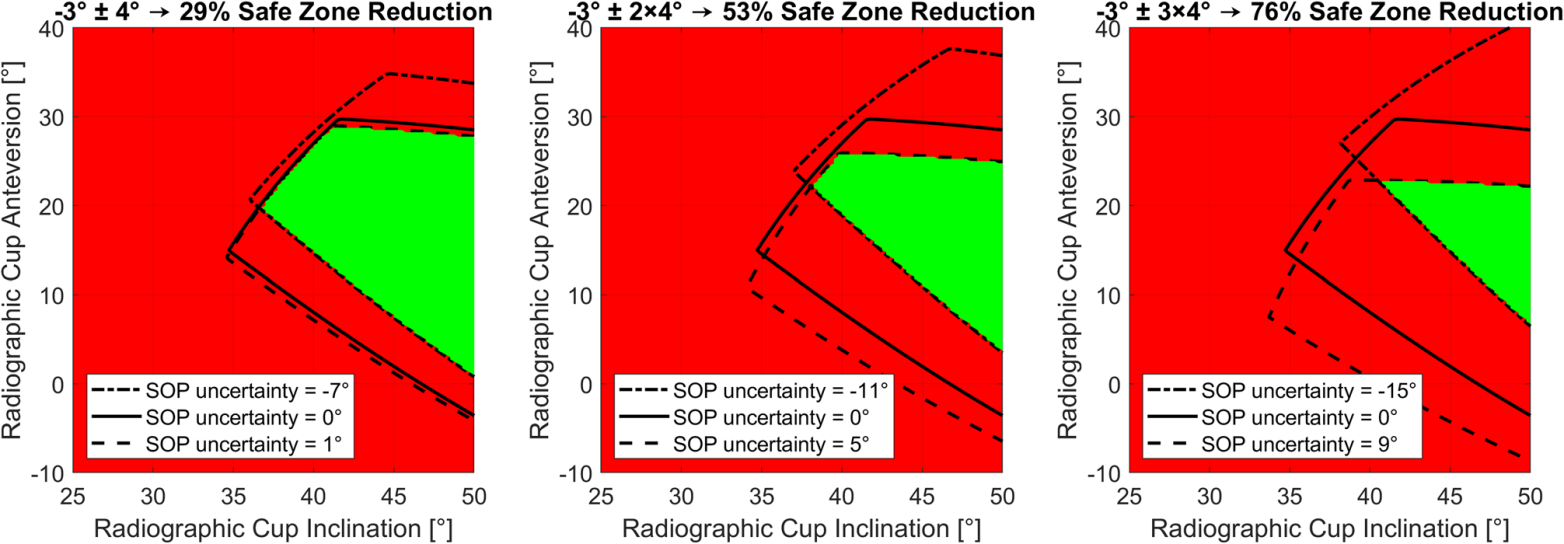
Impact of the uncertainty of the change of the SOP on the range of motion-based safe zone defined by Widmer^46^ calculated for a head diameter of 32 mm, neck diameter of 11.5 mm, CCD angle of 130° and stem anteversion of 15°. The radiographic cup inclination and anteversion relate to the pelvic bone coordinate system based on the APP.

This study supports the finding of previous observations^25,30,45^ that, on average, the postoperative standing SOP in Japanese patients changes significantly in the posterior direction after THA. This change is not significantly affected by the reference plane used to define the SOP. The clinical significance of a change of the standing SOP of − 3° ± 4° can be shown by the impact of the SOP on the size of current patient-specific safe zones based on the range of motion. A safe zone contains the cup orientations in relation to the pelvis that enable a predefined range of motion, free of prosthetic impingement. The size and position of the safe zone depend on several parameters including the SOP. An uncertainty of the change between the preoperative and postoperative SOP has to be taken into account by narrowing the safe zone to avoid postoperative impingement. Figure 2 shows how the safe zone has to be reduced to allow for the uncertainty of the change of the SOP. The mean ± 1SD, ± 2SD and ± 3SD are considered to cover about 68%, 95% or 100% of the patients.

The range of motion-based safe zone defined by Widmer^46^ has to be reduced by 29% for an uncertainty of − 3° ± 4°, by 53% for an uncertainty of − 3° ± 2 × 4° and by 76% for an uncertainty of − 3° ± 3 × 4°. Other postures with a different SOP, such as sitting, or the surgical precision are not considered in this example and could additionally reduce the safe zone. Therefore, Imai et al. consider a measurement error of the SOP of ± 2.5° as a critical threshold^27^.

This study showed that the change of the SOP is already present after 3 months, whereas other studies of Japanese patients investigated the change after one year or later^25,30,45^. Previous studies reported that the posterior change increases over five^47^ and ten years^45^ after THA in Japanese patients. By contrast, a majority of studies outside Japan reported that, on average, there is no significant change between the preoperative and postoperative standing SOP three months^48^, one year^49^ or three years^19^ after THA (Table 6).

**Table 6:** Change of the preoperative to postoperative standing SOP after THA.

Year	Author	n	∅ Age	Country	Follow-up	Method	Evaluation	Ref. plane	Change [°]	*p* value
2003	Nishihara^24^	74	56	Japan	1 year	CT model/AP radiograph matching	Semi-automatic	APP	− 2 (− 7.5, − 26 to 15)	[< 0.05]
2006	DiGioia^1^	84	62	US-PA	3 months	lateral radiograph	Manual	APP	NA	NS
2009	Blondel^19^	50	64	France	3 years	lateral radiograph	Manual	APP	NA	NS
2009	Parratte^2^	21	?	US-MN	2 months	infrared tracking system		SISP	− 1.5 (3.3, − 7 to − 0.6)	NS
					1 year				− 3 (5.3, − 12.8 to 0.4)	< 0.05
2012	Taki^30^	86	64	Japan	1 year	AP radiograph/pelvic cavity regression	Manual	SPPS	3.9 (6.2, NA)	< 0.05
					2 years				5.3 (7.1, NA)	< 0.05
					3 years				5.1 (5.7, NA)	< 0.05
					4 years				5.2 (6.2, NA)	< 0.05
2013	Murphy^26^	30	60	US-MA	1 year	CT model/AP radiograph matching	Semi-automatic	APP	0.5 (3, − 5 to 7.2)	NS
2013	Kyo^25^	124	66	Japan	1 year	CT model/AP radiograph matching	Semi-automatic	APP	− 3.1 (3.6, − 13 to 5)	[< 0.001]
2015	Maratt^8^	138	57	US-NY	1.5 months	lateral radiograph	Manual	APP	− 0.3 (3.6, − 18.4 to 15.0)	NS
2016	Weng^49^	69	63	China	1 year	lateral radiograph	Manual	PTP	NA	NS
								SSP	NA	NS
2016	Suzuki^47^	77	64	Japan	5 years	CT model/AP radiograph matching	Semi-automatic	APP	NA	[< 0.01]
2017	Barbier^48^	40	65	France, Belgium	3 months	lateral EOS image	Manual	PTP	NA	NS
								SSP	NA	NS
2017	Tamura^45^	101	55	Japan	1 year	CT model/AP radiograph matching	Semi-automatic	APP	NA	< 0.05
					5 years				NA	< 0.05
					10 years				− 11.4 (13.2, − 45 to 15)	< 0.05
2018	Berliner^50^	144	61	US-NY	1 year	lateral EOS image	Manual	SSP	NA	< 0.05

n = number of subjects, Ref. plane = reference plane, AP = anterior–posterior, NA = not available, NS = not significant, *p* values in square brackets were calculated by the authors of this study.

Different sample sizes, methodologies or cultural differences might be possible reasons to explain this discrepancy. The Japanese studies mainly used a matching technique between a patient-specific surface model of the pelvis reconstructed from supine CT images (CT model) and a standing anterior–posterior (AP) radiograph to measure the standing SOP, whereas the studies outside Japan mainly used a standing lateral radiograph. A very good reproducibility and reliability was reported for a 3D/2D matching method between the CT model and AP radiograph used by Murphy et al.^26,51^. It seemed reasonable to assume that the combined use of the orthogonal AP and lateral EOS images in the matching process should deliver at least equivalent results, also considering the absence of vertical distortion in the EOS images in contrast to the AP radiograph. The current study introduced a 3D/2.5D registration method (CT2EOS) between the CT model and the biplanar EOS images in the standing position (Fig. 1). The CT2EOS method proved to be superior to the proprietary sterEOS software of the EOS imaging system in a first evaluation using phantom data. The sterEOS software reconstructs the 3D geometry of the pelvis by morphing a generic surface model to manually selected landmarks in the EOS images. However, CT imaging is still the gold standard to reconstruct the 3D geometry of the pelvis. Unfortunately, the EOS software does not support the import of the CT model of the individual patient for the registration with the EOS images. Therefore, the CT2EOS method was developed to determine the position of the pelvis in the standing position since the individual CT model was available for all subjects. If the individual CT model was not available, the 3D reconstruction of the pelvis by a morphed generic surface model, as performed by the sterEOS software, might be a promising alternative.

Imai et al. reported that the measurement of the SOP on standing lateral radiographs is reliable if the measurement is performed once by two observers^27^. However, the SOP on lateral radiographs was only measured once by one observer in some of the studies cited in Table 6^1,8^.

Le Huec et al. reported that the standing SOP in healthy subjects is not significantly different between Japanese and Caucasian subjects using lateral EOS images^52^. Diebo et al. reported that Japanese patients had a significantly different SOP compared to US patients, independent of their adult spinal deformity classification^53^. However, most previous studies that investigated the SOP in the context of THA stated that the inter-subject variability is high. This finding can be confirmed by the results of this study.

Consequently, regression analysis was performed to identify important preoperative patient-specific parameters that improve the prediction of the postoperative standing SOP in Japanese patients. This study showed that the consideration of the preoperative standing SOP and LLA in combination with the preoperative supine SOP improves the prediction of the postoperative standing SOP significantly (Scenario B). Age is also an important factor. However, age on its own in combination with the preoperative supine SOP (Scenario A) cannot substitute the two preoperative standing parameters. This finding is supported by Kyo et al., who identified the age and lumbar alignment as significant predictors for the change of the preoperative supine to postoperative standing SOP^25^. Unfortunately, a direct comparison of the results is not possible since Kyo et al. dichotomized the continuous response variable change of SOP^25^ and performed a logistic regression. This might provide an easier interpretation of the results. However, the dichotomization of continuous variables creates multiple problems including the comparability of the results and is generally not recommended^54,55^. The concept of a unified safe zone for all patients has been questioned and a patient-specific target zone has been proposed in the last decade^5,9,56,57^. In this context, the division of the patients into two or multiple subgroups based on the SOP or change of SOP is questionable and, instead of choosing a more or less unrelated threshold such as 10°^25,47,58^, dichotomization should be avoided.

Sex, weight and BMI did not significantly improve the prediction of the postoperative standing SOP. However, due to the sex ratio of 0.174 males/females, this has to be investigated further. Consequently, the data of more male patients would be necessary. Unfortunately, the common sex ratio of Japanese primary THA patients is 0.198 males/females^59^.

Morphological parameters, such as pelvic incidence, did not improve the prediction of the postoperative standing SOP significantly. This is in accordance with similar studies that did not find a significant correlation between the pelvic incidence and change of the SOP^8^, lumbar-pelvic-femoral kinematics^50^ or an unfavorable pelvic mobility^60^ in THA patients. In this context, the use of the pelvic incidence as a classification factor for patient subgroups in a recently presented cup alignment technique^22^ should be questioned.

The preoperative range of motion from the Harris Hip Score as a measure for preoperative contractures and the check for L5-S1 fusion did not significantly improve the prediction of the postoperative standing SOP in this study. However, only 8% of the patients had a L5-S1 fusion and patients with a fusion of more than two lumbar vertebrae were excluded from this study. These results, therefore, can only be an indication of the effect of an isolated L5-S1 fusion on the postoperative standing SOP. A conclusion about the impact of a fusion of multiple lumbar vertebrae on the postoperative standing SOP cannot be drawn from this study. Multiple studies reported that lumbar fusion increases the risk of dislocation in THA^4^.

Some additional limitations should be considered. Patients were positioned in the shifted feet position in the EOS system. However, Chaibi et al. reported that the shifted feet position did not have a significant effect on the measurement of the SOP^61^. Aside from that, in this study, the 3D orientation of the pelvis was decomposed to adjust the SOP for the transverse and the frontal orientation of the pelvis. The range of motion of the contralateral hip was not evaluated preoperatively. Contralateral contractures might reduce the postoperative change of the standing SOP. No EOS images in the sitting position or in other functional positions of daily living were taken of the patients, thus, preventing the consideration of the sitting SOP in the study. The sitting SOP was identified as an important factor of the patient-specific functional cup orientation^15^. Therefore, the preoperative workflow was recently adapted and new patients receive additional EOS imaging in the sitting position. Moreover, instead of taking lumbar radiographs in combination with lower body EOS images, new patients receive EOS imaging including the lumbar spine.

## Conclusion

A patient-specific prediction of the postoperative pelvic-femoral kinematics during activities of daily living for optimal functional component alignment seems to be a reasonable goal, as it has a direct impact on the prediction and simulation of edge-loading, wear, impingement and dislocation^56^.

A high inter-subject variability of postoperative SOP seems undisputable. This study confirmed that the mean SOP in the standing position in Japanese patients changes significantly after THA and that this change is already present three months after surgery. Further studies need to be carried out to investigate a postoperative change of the SOP. However, in the context of a patient-specific functional cup alignment, the dichotomization of the continuous variable SOP or change of SOP should be avoided. The study highlights the importance of age, lumbar alignment and pelvic-mobility as preoperative factors for the prediction of the postoperative standing SOP. The findings of this study suggest that the consideration of the factor age can improve the prediction of the postoperative standing SOP from the preoperative supine SOP (Scenario A). However, if possible, the preoperative standing SOP and LLA should be measured and considered in addition to the preoperative supine SOP (Scenario B), since they significantly improve the prediction of the postoperative standing SOP. This study did not find a significant improvement of the postoperative standing SOP prediction by the factor pelvic incidence. Further research is required to establish the rationale of considering pelvic incidence in functional cup alignment.

## Supplementary information

Supplementary Information 1.

Supplementary Information 2.

## Data Availability

The datasets generated during and/or analyzed in this study are available from the corresponding author upon reasonable requests.
