# Supplementary material for: Preoperative factors improving the prediction of the postoperative sagittal orientation of the pelvis in standing position after total hip arthroplasty
Source: Sci Rep. 2020 Sep 29;10:15944. doi: 10.1038/s41598-020-72782-1 (PMC7524714; doi:10.1038/s41598-020-72782-1)
Supplement: Supplementary file 1 — Supplementary Information 1. [file 41598_2020_72782_MOESM1_ESM.docx]

Preoperative factors improving the prediction of the postoperative sagittal orientation of the pelvis in standing position after total hip arthroplasty

Supplementary Text S1 – Definitions of the Biometric, Morphologial and Functional Parameters

Maximilian C. M. Fischer¹, Kunihiko Tokunaga², Masashi Okamoto³, Juliana Habor¹, Klaus Radermacher¹

¹Chair of Medical Engineering, Helmholtz-Institute for Biomedical Engineering, RWTH Aachen University, Germany

²Niigata Hip Joint Center, Kameda Daiichi Hospital, Niigata City, Japan

³Department of Radiology, Kameda Daiichi Hospital, Niigata City, Japan

2020

# Biometric parameters

1. Sex: Male or female
2. Age: Preoperative age in years
3. Height: Preoperative body height in cm.
4. Weight: Preoperative body weight in kg.
5. Body mass index (BMI): Weight / (Height / 100)² in kg/m².

# Morphological parameters

## Landmarks

Table 1: Used landmarks. In the text, an "s" at the end of the abbreviation indicates the plural form.

| **Abbreviation** | **Name** |
| --- | --- |
| ASIS | anterior superior iliac spine |
| HJC | hip joint center |
| IS | ischial spine |
| PS | pubic symphysis |
| PS2HJCs | Projection of the PS on the line connecting the HJCs |
| PSIS | posterior superior iliac spine |
| SC | sacral center, center of the sacral plateau |
| SP | sacral promontory |

## Distances in mm

1. ASIS HJC Distance (ASIS-HJC): Distance between the midpoint of the ASISs and the PS2HJCs.
2. PS HJC Distance (PS-HJC): Distance between the PS and PS2HJCs also known as pubic prow^1^.
3. IS HJC Distance (IS-HJC): Distance between the midpoint of the ISs and the PS2HJCs.
4. PSIS HJC Distance (PSIS-HJC): Distance between the midpoint of the PSISs and the PS2HJCs.
5. SC HJC Distance (SC-HJC): Distance between the SC and the PS2HJCs also known as pelvic thickness^1,2^.
6. ASIS PSIS Distance (ASIS-PSIS): Distance between the midpoint of the ASISs and the midpoint of the PSISs.
7. ASIS SC Distance (ASIS-SC): Distance between the midpoint of the ASISs and the SC.
8. ASIS IS Distance (ASIS-IS): Distance between the midpoint of the ASISs and the midpoint of the ISs.
9. PS PSIS Distance (PS-PSIS): Distance between the PS and the midpoint of the PSISs.
10. PS SC Distance (PS-SC): Distance between the PS and the SC also known as sacropubic thickness.
11. PS IS Distance (PS-IS): Distance between the PS and the midpoint of the ISs.
12. PSIS SC Distance (PSIS-SC): Distance between the midpoint of the PSISs and the SC.
13. IS SC Distance (IS-SC): Distance between the midpoint of the ISs and the SC.

## Reference planes

Five different reference planes to measure the sagittal orientation of the pelvis (SOP) were selected from literature.

1. Anterior pelvic plane (APP): The plane defined by the PS and the ASISs.
2. Pelvic tilt plane (PTP): The plane defined by the SC and the HJCs.
3. Sacral slope plane (SSP): The plane defined by sacral plateau.
4. Superior iliac spine plane (SISP): The plane defined by the left and the right ASIS as well as the midpoint between the left and the right PSIS recommended by the International Society of Biomechanics^3^.
5. Sacral promontory – pubic symphysis plane (SPPS): The plane defined by the vector connecting the SP and the PS as well as the vector connecting the left and the right ASIS.

## Angles in °

1. Pelvisacral angle (PSA): Dihedral angle between the SSP and PTP^4^.
2. Pelvic incidence (PI): 90° minus the PSA^4^.
3. Angle of Prow (AoP): Angle between the PS-HJC and the PS-HJC^1^.
4. Sacral anatomical angle (SAA): Dihedral angle between the APP and the SSP^4^.
5. Sacral pubic angle (SPA): 90° minus the angle between the normal of the APP and the vector connecting the SC and the PS^5^.

# Functional parameters

## Measurement of SOP in °

Measurement of the SOP is based on the pelvic reference planes.

1. SOP_APP_ refers to the vertical. SOP_APP_ is positive in anterior direction and negative in posterior direction.
2. SOP_SISP_ refers to the horizontal. SOP_SISP_ is positive in inferior direction and negative in superior direction.
3. SOP_PTP_ refers to the vertical. SOP_PTP_ is positive in anterior direction and negative in posterior direction. Usually, it is vice versa.
4. SOP_SSP_ refers to the horizontal. SOP_SSP_ is positive in anterior direction and negative in posterior direction.
5. SOP_SPPS_ refers to the vertical. SOP_SPPS_ is positive in anterior direction and negative in posterior direction.

## Lumbar parameters

1. Lumbar lordosis angle (LLA): Angle between tangent lines along the vertebral body superior endplates of L1 and S1 in degree on a lateral x-ray of the lumbar spine. Angle is positive if it opens in anterior direction and negative if it opens in posterior direction. In case of a L5‑S1 fusion or a L5‑S1 ankylosis, the angle was measured between L1 and L5.
2. L5-S1 fusion: Yes or No. Yes in case of a L5‑S1 fusion or a L5‑S1 ankylosis. Determined by inspecting the supine CT images and the lateral lumbar x-rays.

## Range of motion

All angles are measured in steps of 5° and taken from the preoperative Harris Hip Score^6^.

1. Flexion: Maximum flexion.
2. Extension: Maximum extension.
3. Abduction: Maximum abduction.
4. Adduction: Maximum adduction.
5. External rotation: Maximum external rotation.
6. Internal rotation: Maximum internal rotation.

# References

1. Legaye, J. *et al.* Relationship between Sacral Pelvic Incidence and Acetabular Orientation. *Hip international: the journal of clinical and experimental research on hip pathology and therapy* **21,** 87–97; 10.5301/HIP.2011.6283 (2011).

2. Boulay, C. *et al.* Anatomical reliability of two fundamental radiological and clinical pelvic parameters: incidence and thickness. *European journal of orthopaedic surgery & traumatology : orthopedie traumatologie* **15,** 197–204; 10.1007/s00590-005-0239-5 (2005).

3. Wu, G. *et al.* ISB recommendation on definitions of joint coordinate system of various joints for the reporting of human joint motion—part I. Ankle, hip, and spine. *Journal of Biomechanics* **35,** 543–548; 10.1016/S0021-9290(01)00222-6 (2002).

4. Vrtovec, T. *et al.* A review of methods for evaluating the quantitative parameters of sagittal pelvic alignment. *The spine journal : official journal of the North American Spine Society* **12,** 433–446; 10.1016/j.spinee.2012.02.013 (2012).

5. Sautet, P. *et al.* Is anatomic acetabular orientation related to pelvic morphology? CT analysis of 150 healthy pelvises. *Orthopaedics & traumatology, surgery & research : OTSR* **104,** 347–351; 10.1016/j.otsr.2017.10.006 (2018).

6. Harris, W. H. Traumatic arthritis of the hip after dislocation and acetabular fractures: treatment by mold arthroplasty. An end-result study using a new method of result evaluation. *The Journal of bone and joint surgery. American volume* **51,** 737–755 (1969).
